# A Retrospective Review of Patient Records and Factors Associated with Decisions Made by Community Nurse-Paramedics’ in Finland

**DOI:** 10.3390/nursrep11030065

**Published:** 2021-08-31

**Authors:** Tuija Rasku, Mika Helminen, Marja Kaunonen, Elizabeth Thyer, Eija Paavilainen, Katja Joronen

**Affiliations:** 1 Faculty of Social Sciences, Health Sciences, Tampere University, Kuntokatu 3, 33520 Tampere, Finland; Mika.Helminen@tuni.fi (M.H.); Marja.Kaunonen@tuni.fi (M.K.); Eija.Paavilainen@tuni.fi (E.P.); 2 Tays Research Services, Tampere University Hospital, 33520 Tampere, Finland; 3 General Administration, Pirkanmaa Hospital District, Tampere University, 33014 Tampere, Finland; 4 Dean’s Unit School of Health Sciences, Western Sydney University, Locked Bag 1797, Penrith, NSW 2750, Australia; E.Thyer@westernsydney.edu.au; 5 General Administration, The Hospital District of South Ostrobothnia, 60220 Seinäjoki, Finland; 6 Department of Nursing Science, University of Turku, Joukahaisenkatu 3-5, 20520 Turku, Finland; katja.joronen@utu.fi

**Keywords:** nurses, community paramedicine, prehospital care, non-emergency care, decision-making

## Abstract

Community paramedicine (CP) has extended the role of paramedics and the main goal is to provide non-emergency care, which reduces the visits to emergency departments. The aim of this study was to describe the Finnish CP and examine the factors that were involved in CNPs’ decision-making processes. The study was based on data from 450 consecutive CP patient records from three hospital districts. A more detailed analysis was carried out on 339 cases in patients’ homes and elderly care homes, and the data analysis included multivariate logistic regression to examine the impact of variables on the CNPs’ decisions. These patients’ most common health issues were general weakness (15.9%) and fever (10.6%), and over half (58.7%) could remain at home after the CP visit. There were five independent factors associated with the CNPs’ decisions of the patient’s care continuum: the hospital district, if the patient could walk, whether the troponin test was performed, a physician was consulted, and the nature of the task. CP units played a valuable role in non-emergency care. Understanding the factors associated with CNP decision-making can increase the safety and effectiveness of reducing hospital visits, by providing patient care at home, or in elderly care facilities.

## 1. Introduction

In 2018, the World Health Organization (WHO) launched its vision for primary health care pathways that centre on people rather than services (WHO, 2018). Two years earlier, the Organisation for Economic Co-operation and Development (OECD) had called for paramedics and nurse practitioners to play an enhanced role in tackling workforce shortages and delivering more accessible out-of-hours care [1]. Studies have explored how the extended role of paramedics’ has contributed to primary health care [2,3,4,5]. Since 2005, community paramedicine (CP) programmes have undertaken health promotion and illness prevention work at community levels in Australia, Canada, the United States of America, and the United Kingdom. They have filled care gaps and decreased pressure on emergency departments (EDs) by dealing with patients who did not need that level of attention [3,5].

Various CP programmes have been provided by pre-hospital and post-hospital or community health services [3]. Pre-hospital CP services include assessing and treating patients, as needed, and referring or releasing them instead of transporting them to the ED. Many pre-hospital CP programmes address the needs of people who frequently call emergency numbers or visit EDs. [3,4,5]. Post-hospital and community health services are run by CP programmes and provide follow-up care for recently discharged patients. For example, community paramedics have been reported to help patients with self-care of chronic diseases, and worked with community health workers to provide preventive care [4,5,6]. Services provided by CP programmes have also been linked with nutritional assistance programmes or behavioural health services such as screening for depression [7]. Feedback on home-based CP programmes have showed that patients were satisfied that they could receive primary health care close to home, it gave them a sense of security and support, and they felt empowered to enhance their health management [5,8].

In 2017, the Finnish government stated that emergency medical services (EMS) could create single responder units to provide non-emergency patient assessments and provide back-up units for emergency ambulances [9]. These single responder units are staffed with an advanced level nurse-paramedic (NP), or a nurse specialized in prehospital emergency care. Single responder-units are allocated cases by the dispatch centre or can respond directly to the staff that provides care in people’s homes and elderly care homes. In Finland, the call outs from the dispatch centre are prioritized into four categories: A (the patient might have a life-threatening emergency), B (the patient is stable but might have other urgent), C (the patient needs an acute assessment), and D (the patient has a non-emergency situation). The EMS single responder-units are known by various names in different countries and they even differ among hospital districts in Finland. In this study, we use the term CP units, the staff are Community nurse-paramedics (CNP) and the home-care patient (HCP) means the patient at home or in the elderly care homes. 

EMS policies and protocols guide the assessments and decisions made by CNPs, but complex decisions can fall outside the scope of protocols and they have access to physicians for further advice. Halter et al. [10] identified four factors that were considered by EMS staff when they made decisions about patients. They assessed the information provided by the dispatchers before they arrived. On arrival, they carried out an initial assessment to determine whether the patient needed imminent emergency care, or whether it was not an emergency. Medical and social information was gathered from the patient and then, the paramedics decided whether the care they needed could be provided in situ at home or whether they needed to be transported so that they could receive specialist care. The most reported health outcomes of CP programmes have included patients being transported to EDs or patients being admitted to hospitals [5]. The international impact of CP programmes has been described by multiple authors [4,5,7] and the consensus for a standardized patient assessment has been researched [11]. 

Finland is divided into 21 hospital districts, which organize EMS. The Finnish CP units are part of the EMS system. The EMS includes first responders, basic life support units, advanced life support units, and physician-led units. The basic life support unit is staffed by a firefighter or an emergency medical technician, plus a nurse specialized in prehospital emergency care. At least one member of the advanced life support unit team is a paramedic, who is a registered nurse. These nurse-paramedics have either completed a four-year Finnish Bachelor’s degree or have specialized in prehospital emergency care for one year. Both are registered as nurses. The Finnish CP units are staffed by CNPs, who are either advanced level paramedics with many years of experiences or nurses with many years of experiences of emergency care. CNPs also undergo additional training in areas such as advanced diagnostic and medication management. The CP vehicle is equipped in a like to the advanced life support unit, but without a stretcher or immobilization equipment. It does include point-of-care blood testing and 16-lead electrocardiograms. The CP units provide patient assessments, follow-up care, and minor treatments for not-life-threatening emergencies. Sometimes, the CP unit can start the emergency care together with the ambulance units. CNPs can administer broader medication than advanced life support units [12]. In Finland, the patient assessment and care provided by CP units are based on national and regional treatment protocols and the National Health Care Act. They can also call on the expertise of an EMS physician, as required. 

The aim of this CP study was to describe the Finnish community paramedicine and examine the factors associated with CNPs’ decision-making processes. The CP model is a novel care model in Finland and to the best of our knowledge, this was the first study to do this. 

## 2. Materials and Methods

This was a quantitative descriptive study based on a retrospective review of CNPs’ patient records from CP models in three hospital districts in Finland. We used the SQUIRE checklist when writing our report [13].

### 2.1. Data Collection and Measurement

This study focused on three hospital districts in different parts of Finland, who had introduced the CP model in April 2016, January 2017, and March 2018. The districts were a similar size, with populations ranging from 130,000 to 190,000, and each one covered both rural and urban areas. During Spring 2019, the researcher (TR) travelled to each three hospital districts and collected the data from the CP units’ electronic patient record systems. Hair et al. [14] defined reliability as an assessment of the degree of consistency between multiple measurements of a variable and the recommended sample size can be calculated when the number of variables is multiplied with 5–10 observations. Multiple imputations were applied for the missing data of continuous variable [14]. We multiplied the 19 variables and added 17 for any loss ending up to the sample size of 150 CP patient records from each Hospital District. The CP patient records were the first 150 CP patients contacted at the start of the second year of each three CP models. If a patient appeared more than once in the data, then only the first contact was included. 

The information was transferred directly from the patient records to the computerized abstraction form (Table 1). The form was composed using the EMS assessment and decision-making structure from Halter et al. [10] (prearrival information, Initial contact and Continuing assessment and Making a conveyance decision) and the SV210 EMS patient chart from the Finnish Ministry of Social Affairs and Health. The chart comprised five parts: information collected before arrival at the patient’s home or elderly care home or before carrying out a telephone assessment, patient data, information from the patient during initial contact, information from the continuing assessment, and deciding whether an ambulance was needed to transport the patient for further specialist care. The continuing assessment was confirmed by using the International Classification of Primary Care, Second Edition (ICPC-2), which was developed by the World Organization of Family Doctors’ and revised in 2015 [15].

The time of day was divided into the dayshift (9:00 a.m.–8:59 p.m.) and the nightshift (9:00 p.m.–8:59 a.m.). The patient ages were split into four categories (Table 2). The time spent with the patient was measured in minutes. For face-to-face visits, it related to the moment the patient was first seen to leaving their home or elderly care home. For telephone calls, it was the total length of the call. Several variables were dichotomized based on whether they were documented or not; Airway, Breathing, Circulation, Disability, Exposure (ABCDE)-approach, analysed or not; the patients’ blood glucose levels, C-Reactive protein, troponin and prothrombin, and whether an electrocardiogram had been performed and a physician had been consulted. 

### 2.2. Data Analysis

The data were analysed with SPSS statistical software for Windows, release 25 (SPSS, Chicago, IL, USA). Descriptive statistics included frequencies, percentages, means, and medians. Age and contact time were categorized for further analyses. Missing data of the patient’s background, namely gender, age, and the patient’s position when the CNP first saw or spoke to them were treated as independent variables groups. We kept all the variables for the characteristics of the CP patients. The decisions made by the CNPs, including whether a patient remained at home or needed to be transported, were analysed using the chi-square test and described with cross-tabulation. Statistical significance was set at *p* < 0.05.

A regression model was constructed to identify the factors associated with the CNPs’ decision making when the patient remained at home or needed ambulance transportation to the hospital. Univariate logistic regression analysis was applied to screen the association between each associated factor with the CNPs’ decision making. Each of the predictor variables underwent univariable logistic regression analysis, and those that were significant (*p* < 0.05) were included in the multivariable regression model. Multivariate regression analysis was used to estimate the predictors of the CNPs’ decision making. A backward elimination approach was used to fit the final multivariable model. We wanted out model to accurately reflect the key factors of the CP decision-making. Goodness of fit of any final model was tested using Nagelkerke’s R^2^ test. The final model interpreted the ORs, 95% confidence intervals (CI), and *p*-values to estimate the association between the patient care continuum and the independent predictors of CNPs’ decision-making.

### 2.3. Ethical Considerations

The study was approved by Tampere University Hospital Ethics Committee reference number: R19008H) and the participating hospital districts and their EMS managers. We also needed specific permission from the three patient data registrars. Informed patient consent was not required, as the patients were anonymized to guarantee their privacy and confidentiality. The hospital data analysts collected the information from the patients’ records on our behalf, without surnames, birth dates, or addresses. The principal author retrospectively reviewed all the patient data that had been collected. The codes allotted to the patients simply contained a gender code and their birth year. All the data were passed to the first author, handled, and stored in accordance with current legislation and downloaded to Microsoft Excel for Windows 10 (Microsoft Corp, Redmond, WA, USA).

## 3. Results

### 3.1. All CP Cases

The CP units received calls from all sources (home care nurses, dispatch centres, EMS field supervisors, police, families). The most common ICPC-2 codes for all patients (*n* = 450), were acute alcohol abuse (15.6%), general weakness or tiredness (12.4%), fever (8.4%), and chest pain (7.3%). Just under a third of the 450 CP patients (32.4%) needed ambulance transport for further assessment and two-thirds (67.6%) were able to stay at the police station or at home or in their elderly care home (Table 2).

### 3.2. Patients at Home or in Elderly Care Homes 

The analysis was carried out after we excluded 111 cases: 80 were clients from the police, nine back-up duties for other EMS units and 22 further contacts with three terminal patients. The analysis was carried out from those CP patients (*n* = 339, 75.3%) who lived at home or in elderly care homes These home-care patients had a median age of 83 years (min 19–max 103) and more than one-third (38.2%) were 85 years or older. In 39.2% of the cases, the CNP provided telephone triage and the remaining patients were assessed face-to-face (Table 2).

Most of the 187 (51.6%) calls from the patient’s home were from home care nurses or assistants, 1.5% were from personal alarm call workers, and the remainder were the patients or their friends or family. The calls to the CP units were relayed from the EMS central dispatch centre using all the priority codes from A to D. The final 12.1% were referred to the CP units by ambulance units, so that the CNP could continue the patient’s care and free up the ambulances for other calls (Table 2).

The documentation of the patients’ ABCDE assessments varied. The most frequently recorded element was the current medication, which was documented for 226/339 (66.7%) patients. The analysis showed that the median number of medications taken per day was eight (min 0–max 23) and 24 of these 226 patients (10.6%) took more than 20 different medications per day. 

Point-of-care tests were used 159 times during the assessments. The physician was consulted about nearly a third (30.4%) of the 339 patients. The contact time was available for 191/339 (56.3%) of the patients. (Table 2) The median (min–max) contact time with the patients was 31 (1–229) minutes and was considerably higher for telephone contact (5, 1–185) minutes than home attendances (63, 5–229) minutes (Table 2). The most frequent ICPC-2 classifications in the 339 patients were general weakness/or tiredness (15.9%), fever (10.6%), transient cerebral ischemia (8.8%), and chest pain (8.3%). Two of the patients were classified with social problems (Table 3). 

### 3.3. Factors Associated with the CNPs’ Decision 

The univariate analyses indicated 14 predictors that were related to the CNPs’ decisions about the patients’ care continuum. Having received initial information about the patients, the CNP assessed 133/339 (39.2%) of the patients by phone and in 65 (48.9%) of those cases, an ambulance unit was sent to carry out a further assessment. In the other 68 cases, the patient was able to remain at home. Only seven (15.2%) of the patients, who were able to walk when the CNP arrived, needed to be transported to the hospital by ambulance. There were no statistical differences between the days of the week (*p* = 0.084) (Table 4).

Five predictors were still statistically significant after the multivariable logistic regression analysis and these were hospital district, patient position when first contact was made, troponin test performed, consulting a physician, and nature of the task. Patients had a higher probability of remaining at home in hospital district three (OR 5.5, 95% CI 2.77–11.2, *p* < 0.001) than in hospital district one. The patients’ odds for remaining at home were higher (OR 11.6, 95% CI 1.2–110.9, *p* = 0.034) when the CNP provided the troponin test or (OR 3.1, 95% CI 1.7–5.5) consulted the physician. The patient’s odds for remaining at home was higher (OR 3.9, 95% CI 1.4–11.0, *p* = 0.029) when the CNPs’ nature of task included assessment and treatment, compared to patient assessment only (Table 5).

## 4. Discussion

The aim of this CP study was to describe the Finnish community paramedicine and the factors associated with CPNs’ decision-making. To the best of our knowledge, this was the first study to do that.

The Finnish CP models varied in each Hospital District. However, all three CP models served a population of all ages (range 19–103). The median age of the patients at home or in elderly care homes was high: 83 years. Globally, older patients have been the main population served by 48.4% of CP programmes [6]. Some CP programmes have been used in the wider community [16]. Others have focused on patients with congestive heart failure or diabetes [7], or provided regular visits to older people in subsidized housing [17]. The Finnish CP models could be adapted to provide more tailored programmes that engage with special patient groups as well as the community as a whole.

The CNPs assessed the patient’s care need by phone or during the visit. In our study, the care needs of 133 (39.2%) of the home care patients were assessed by phone and less than half (48.9%) of them needed a further assessment by an ambulance unit. Phone assessment could play a considerable role in assessing and managing non-emergency cases and this has been particularly evident in many aspects of primary health care during the current pandemic. Telemedicine can also help medical staff to reach patients who live some distance from emergency services, but it cannot provide a substitute for face-to-face communication and safe care backed up by other collaborative services.

In this study, advanced diagnostics were a novel part of the assessment and treatment. The troponin and CRP test formed part of the need for follow-up care. Research has shown that point-of-care tests have been a reliable part of the CNP assessment and that critical range discrepancies occurred in less than 1% of cases compared to laboratory tests [18]. Point-of-care tests also detected cardiac damage in 91% of prehospital acute myocardial infarction-patients [19] and the CRP test has been considered an important tool in assisting the care continuum of patients [20]. The CP model means that patients were able to avoid visiting hospitals for one blood test or for the control test, because they could receive the care they needed at home.

Our study results indicate that being able to consult physician had a positive impact on decisions. This contrasted with a previous study, which found that consulting an on-call physician was not associated with whether CP patients were transported to the hospital [12]. Leikkola et al. [21] concluded that consulting physician could be challenging if the physician did not concentrate sufficiently on the phone call or enter the background information that care providers requested into the patient’s records. However, the physicians in our study appear to have provided information that enabled the patients to stay at home. The CNPs could discuss treatments and care planning and confirm with the physician that the patients could receive the care they needed at home.

We found that the most common patient ICPC-2 classification was general weakness or tiredness, which was consistent with a previous EMS study [22]. In addition, the high number of medications could point to patients having multiple morbidities. The increased complexity of prehospital care that some patients need has also been highlighted by previous research [23]. One notable finding was that patients’ nonspecific complaints or general weakness increased mortality [24], which could increase the challenge of finding patients the right care in the right place. The variety of complaints and multi-morbidities of the CP patients in our study showed the wide-ranging competencies that CNPs need when assessing and progressing a patient’s care.

More than half (58.7%) of these 339 patients received the care and treatment that they needed at home or in their elderly care home. This percentage was higher than a previous, comparable study, where 48% of the patients stayed at home after they were visited by a CP unit [12]. One international study reported that the 84.6% of CP programmes used reduced ED visits as an outcome measurement [7]. Another stated that patients found being transported to the ED very stressful [8], thus being able to avoid this could have a positive impact on the psychosocial well-being of patients’ and their families. Furthermore, being able to remain at home can help patients avoid hospital acquired infections, which is particularly pertinent during the COVID-19 pandemic.

Based on our findings, the CNPs were more likely to organize transport to hospital during dayshifts than nightshifts. Previous studies have reported that it was better to postpone treatment or an ED visit for a few hours, until it was daylight, than transport the patient there during the night [21,25]. Some hospital districts only operate the CP model during office hours. However, our results underlined the importance of the CP units being available day and night, as they often needed to fill the health-care gap when home nursing was not available during the night or at weekends.

According to Halter et al. [10], EMS staff receive prearrival information from the Dispatch Centre. In our study, mainly (55.2%) the CP calls came direct from patient’s home and the CNP needs to triage the patient’s care need immediately. The CNP does not have the arrival time or a team member to discuss about the different options for the patient’s care continuum. The results in our study underlined the medical focus that drove the CNPs’ initial contact and continuing assessment, consistent with the results from Halter et al. [10]. However, the CP providers were also in a unique position to observe and assess the risks and care needs of patients based on their social and environmental determinants of health [8], as Halter et al. [10] has also highlighted.

One of the strengths of this study is that it provides useful information from CP models in three different parts of Finland. The characteristics of the cases were carefully reviewed and abstracted into a specific form. The patients’ signs and symptoms were classified with ICPC-2 codes, and this provided detailed information about their clinical care needs. Factors associated with the CNPs’ decision were defined and the multivariate logistic regression analysis provided a declarative model to explore more in further studies. The study was conducted with an experienced research group, and a statistician was consulted.

The study has some limitations, including the retrospective design, and the fact that the registries containing the patient records were not primarily designed for scientific research. Some information was missing on several patients and further research is needed to find out why this occurred. However, we do know that most of the patients with missing documentation were transported to hospital. It may be that the CNP passed the call to the ambulance unit with any information gathered and did not consider that recording two sets of data was useful. According to Porter et al. [26], EMS professionals do not see that their documentations have significant importance, which can have a general impact on the quality of documentation. However, detailed patients’ records are an essential part of quality management. We did not assess the patients’ follow-up care or outcomes, which would have indicated whether it was right to leave patients at home. Finally, we had three terminal patients that accounted for 22 of the 339 calls, but we only included the first call in our results to avoid skewing the data. Despite these limitations, this study provides a reflection of CP models in Finland and detailed factors associated with the CNPs’ decisions, which will be useful for further research.

These results are nationally and internationally important for those who develop and lead EMS systems and primary health care and for those who educate the healthcare providers of the future. Future research will need to identify the patient groups that would benefit the most from CP services, how these services could engage with the local community, and how the CNPs consider their role and challenges with decision-making.

## 5. Conclusions

This study focused on the work of three CP models in three Hospital Districts in Finland. It describes the five key factors associated with decisions made by CNPs when they assessed home care patients. We believe that the findings from these three CP models could help answer calls from the WHO and OECD for more patient-centered care to be provided at home. This would also enable emergency response services and hospital emergency departments to focus on those patients who have the greatest need for urgent, specialist care.

## Figures and Tables

**Table 1:** The variables involved in the CNPs’ decision-making process.

**The patient** 1. Hospital district 2. Gender 3. Age 4. Medication **Prearrival information** 5. Weekday 6. Time of day 7. Origin of the call 8. Triage code from EMS Dispatch Centre 9. Priority level from EMS Dispatch Centre	**Initial contact and Continuing assessment** 10. Contact 11. Patient position 12. Airway–Breathing–Circulation–Disability–Explore documented 13. Point-of-care test taken for analysis 14. Electrocardiogram performed 15. Physician consulted 16. Nature of the task 17. International Classification of Primary Care-2 18. Contact time Making a conveyance decision 19. Patient able to remain at home or in elderly care home or needed ambulance transport to the emergency department

**Table 2:** Demographic characteristics of the 339 CP patients.

	Missing	%	(*n*)		Missing	%	(*n*)
**Hospital district**				**Patient’s gender**	49		
One		27.1	(92)	Female		58.3	(169)
Two		29.2	(99)	Male		41.7	(121)
Three		43.7	(148)	**Patient’s age**	54		
**Medication** ≤5 6–10 11–19	113	9.7 31.4 48.2	(22) (71) (109)	Under 64 years 65–74 years 75–84 years Over 85 years		15.8 16.1 29.8 38.2	(45) (46) (85) (109)
**≥20**		10.6	(24)	**Origin of the call**		38.2	(109)
**Time of Day** Dayshift 9:00 a.m.–8.59 p.m. Nightshift 9:00 p.m.–8:59 a.m.		79.1 20.9	(268) (71)	From patient’s home From Dispatch Centre From Ambulance Unit		55.2 32.7 12.1	(187) (111) (41)
**Day**				**Triage code**	221		
Monday		13.6	(46)	774A-D Weakness		16.0	(54)
Tuesday		13.3	(45)	706A-B Stroke		6.5	(22)
Wednesday		15.3	(52)	704 A-D Chest pain		3.3	(11)
Thursday		14.2	(48)	745B-D Fallen down		2.4	(8)
Friday		14.7	(50)	783D Backache		1.8	(6)
Saturday Sunday		12.7 16.2	(43) (55)	703B-C Breathing Other triage codes		1.5 3.6	(5) (12)
**The contact**				**Patient position**	21		
By phone		39.2	(133)	Walking		14.5	(46)
By visiting		60.8	(206)	Sitting		26.1	(83)
**ABCDE-approach**				in bed		59.4	(189)
Airway, Breathing		70.5	(239)	**Test analysed**			
Circulation		77.9	(264)	Blood glucose test		19.5	(66)
Disability		79.6	(270)	C-Reactive protein test		19.5	(66)
Explore		38.3	(130)	Troponin test		3.5	(12)
**Electrocardiogram** taken		11.2	(38)	Prothrombin time test		4.4	(15)
**Contact time**	148			**Physician** consulted		30.4	(103)
under 10 min 11–30 min 31–60 min 61–120 min over 121 min		25.7 24.1 15.2 29.3 5.8	(49) (46) (29) (56) (11)	**Nature of the task** Patient’s assessment Assessment and treatment As a Back up-unit **CNPs’ decision**		81.7 9.1 9.1	(277) (31) (31)
				Patient remained at home		58.7	(199)
				Patient needed ambulance transportation		41.3	(140)

**Table 3:** Main categories in which 339 CP home-care patients were placed based on ICPC-2.

ICPC-2 Main Category	*n*	%	ICPC-2 Main Category	*n*	%
A General and unspecified	158	46.5	R Respiratory	28	8.3
B Blood, blood forming organs	2	0.6	S Skin	8	2.4
D Digestive	22	6.5	T Endocrinology	7	2.1
K Cardiovascular	52	15.2	U Urological	6	1.8
L Musculoskeletal	24	7.1	Y Male genital	1	0.3
N Neurological	21	6.2	Z Social Problems	2	0.6
P Psychological	8	2.4			
Total				339	

**Table 4:** Univariable logistic regression of predictors of CNPs’ decisions of patient at home (*n* = 339).

Characteristics	Patient Could Remain at Home % (*n*)	Patient to the Hospital by Ambulance % (*n*)	Unadjusted OR (95% CI) *	*p*
**Hospital district**				<0.001
One	38.0 (35)	62.0 (57)	1	
Two	52.5 (52)	47.5 (47)	1.8 (1.0–3.2)	
Three	75.7 (112)	24.3 (36)	5.1 (2.9–8.9)	
**Patient’s gender**				0.001
Male	59.5 (72)	40.5 (49)	1	
Female	65.1 (110)	34.9 (59)	1.3 (0.8–2.1)	
Missing	34.7 (17)	65.3 (32)	0.4 (0.2–0.7)	
**Patient’s age**				0.003
Under 64 years	62.2 (28)	37.8 (17)	1	
65–74 years	60.9 (28)	39.1 (18)	1.0 (0.4–2.3)	
75–84 years	62.4 (53)	37.6 (32)	1.0 (0.5–2.0)	
over 85 years	66.1 (72)	33.9 (37)	1.2 (0.6–2.5)	
Missing	33.3 (18)	66.7 (36)	0.3 (0.1–0.7)	
**Day**				0.084
Monday	47.8 (22)	52.2 (24)	0.8 (0.4–1.8)	
Tuesday	64.4 (29)	35.6 (16)	1.6 (0.7–3.7)	
Wednesday	53.9 (28)	46.1 (24)	1.1 (0.5–2.2)	
Thursday	77.1 (37)	22.9 (11)	3.0 (1.3–7.1)	
Friday	54.0 (27)	46.0 (23)	1.1 (0.5–2.3)	
Saturday	62.8 (27)	37.2 (16)	1.5 (0.7–3.4)	
Sunday	52.7 (29)	47.3 (26)	1	
**Time of Day** Dayshift 9:00 a.m.–8.59 p.m. Nightshift 9:00 p.m.–8:59 a.m.	56.0 (150) 69.0 (49)	44.0 (118) 31.0 (22)	1 1.8 (1.0–3.1)	0.050
**Origin of the call** From Home From Ambulance Unit From EMS Central Dispatch Centre	66.3 (124) 56.1 (23) 46.9 (52)	33.7 (63) 43.9 (18) 53.1 (59)	1 0.7 (0.3–1.3) 0.5 (0.3–0.7)	0.004
**The contact** By phone By visit	51.1 (68) 63.6 (131)	48.9 (65) 36.4 (75)	1 1.7 (1.1–2.6)	0.023
**Patient position** in bed Walking Sitting Missing	47.1 (89) 84.8 (39) 74.7 (62) 42.9 (9)	52.9 (100) 15.2 (7) 25.3 (21) 57.1 (12)	1 6.3 (2.7–14.7) 3.3 (1.9–5.9) 0.8 (0.3–2.1)	<0.001
**ABCDE-approach**				
**AB** Not documented/documented	66.4 (93)/73.4 (146)	33.6 47/26.6 (53)	1/1.4 (0.9–2.2)	0.169
**C** Not documented/documented	45.3 (34)/62.5 (165)	54.7 (41)/37.5 (99)	1/2.0 (1.2–3.4)	0.008
**D** Not documented/documented **E** Not documented/documented	43.5 (30)/62.6 (169) 42.3 (103)/73.9 (96)	56.5 (39)/37.4 (101) 50.7 (106)/26.1 (34)	1/2.2 (1.3–3.7) 1/2.9 (1.8–4.7)	0.004 <0.001
**Troponin-test** Not performed/performed	57.5 (188)/91.7 (11)	42.5 (139)/8.3 (1)	1/8.1 (1.0–63.7)	0.046
**Electrocardiogram** Not performed/performed	56.5 (170)/76.3 (29)	43.5 (131)/23.7 (9)	1/2.5 (1.1–5.4)	0.023
**Physician consulted** No/yes	52.1 (123)/73.8 (76)	47.9 (113)/26.2 (27)	1/2.6 (1.6–4.3)	<0.001
**Nature of the task** Patient´s assessed Patient assessed and treated As a Back Up unit	59.6 (165) 77.4 (24) 32.3 (10)	40.4 (112) 22.6 (7) 67.7 (21)	1 3.4 (1.5–8.0) 0.5 (0.2–1.0)	0.003

* OR = Odds Ratio; CI = confidence interval.

**Table 5:** Multivariable logistic regression of predictors of CNPs’ decisions of patient at home.

Factors	Adjusted OR (95% CI) *	*p*-Value
Hospital district		<0.001
One	1	
Two	1.9 (1.0–3.9)	
Three	5.5 (2.7–11.2)	
Patient position		<0.001
In bed	1	
Walking	7.2 (2.9–18.1)	
Sitting	2.7 (1.5–5.1)	
Not documented	2.9 (1.0–8.2)	
Troponin test		0.034
Not performed	1	
Performed	11.6 (1.2–110.9)	
Physician consulted		<0.001
No	1	
Yes	3.1 (1.7–5.5)	
Nature of the task		0.029
Patient assessed	1	
Patient assessed and treated	3.9 (1.4–11.0)	
Back Up at patients’ home	0.8 (0.3–2.1)	

* OR = Odds Ratio; CI = confidence interval. Gender, age, time of day, who called, the contact, documented circulation, disability, or explore, and performed electrocardiogram were controlled as independent variables in the model, but they were not statistically significant.

## Data Availability

The literature sources used to contribute to our conclusions are outlined within the manuscript. The data from the patients’ records are not made publicly available due to them containing information that could risk our participants’ confidentiality.
